# Preparation of ginsenoside Rg3 liposomes and evaluating its protective effect on models of Parkinson’s disease

**DOI:** 10.3389/fcell.2026.1963330

**Published:** 2026-10-21

**Authors:** Xiaohan Zhang, Zhuohui Zhang, Yuan Du, Xue Jia, Rongcai Liang, Bing Han, Fenghua Fu, Tian Wang

**Affiliations:** 1 Center for Mitochondria and Healthy Aging, College of Life Science, Yantai University, Yantai, Shandong, China; 2 School of Pharmacy, Key Laboratory of Molecular Pharmacology and Drug Evaluation, Ministry of Education, Collaborative Innovation Center of Advanced Drug Delivery System and Biotech Drugs in Universities of Shandong, Yantai University, Yantai, Shandong, China

**Keywords:** anti-apoptosis, antioxidant, ginsenoside Rg3, liposomes, neuroprotection, Parkinson’s disease

## Abstract

Ginsenoside Rg3 (Gs-Rg3), a bioactive component of *Panax ginseng* C. A. Mey., shows neuroprotective potential in Parkinson’s disease (PD). However, the therapeutic efficacy of Gs-Rg3 is constrained by its poor water solubility and low oral bioavailability. This study aimed to develop ginsenoside Rg3 liposomes (Rg3-Lips) and investigate their neuroprotective effects and mechanisms on PD models. Rg3-Lips were prepared by the thin-film dispersion method. *In vitro*, SH-SY5Y cells were exposed to 1-methyl-4-phenylpyridinium (MPP^+^) and treated with Rg3-Lips. Cell viability, reactive oxygen species (ROS), apoptosis, molecules in Nrf2/GCLM and Bcl-2/Bax/Caspase-3 pathways were assessed. *In vivo*, PD mice were treated with Rg3-Lips or Gs-Rg3. Motor functions were evaluated and dopaminergic nerve, apoptosis, GSH, and relevant proteins in substantia nigra and striatum were analyzed. Optimized Rg3-Lips exhibited a mean particle size of 170.78 nm, an encapsulation efficiency of 93.33%, and a zeta potential of −1.92 mV. *In vitro*, Rg3-Lips significantly increased cell viability, reduced LDH release and ROS levels, upregulated levels of nuclear Nrf2, GCLM and downregulated Cleaved Caspase-3 expression. *In vivo*, Rg3-Lips improved motor functions of PD mice, increased TH-positive neurons and fibers in the substantia nigra and striatum, reduced apoptosis, elevated GSH levels, and modulated Nrf2/GCLM and Bcl-2/Bax/Caspase-3 protein expression, showing better efficacy than Gs-Rg3. In summary, Rg3-Lips exerted neuroprotective effects in experimental PD models, suggesting its potential neuroprotective and therapeutic effects in experimental PD models. Furthermore, Rg3-Lips led to improved pharmacological effects over the conventional active pharmaceutical ingredient.

## 1 Introduction

Liposomes are spherical vesicles with a membrane composed of a phospholipid bilayer, whose structure is like that of biological cell membranes. The characteristic of liposome (e.g., size, amphiphilic character, biocompatibility) makes them be widely used as drug carriers. As a drug delivery system, liposomes can elevate solubility and stability of compounds. Liposomes encapsulate both hydrophilic and hydrophobic drugs, which will improve the solubility of poorly water-soluble compounds, increasing their bioavailability (Partoazar et al., 2025). Liposomes reduce the off-target toxicity of drugs and improve medication safety. It also optimizes pharmacokinetic profiles and then increases the drug concentration in the targeted location. Up to date, many liposomal drugs have obtained clinical application approval from FDA, such as Doxil, Vyxeos, Exparel and Arikayce (Shah et al., 2020).

Most natural products are liposoluble with low oral bioavailability, which limits their development into drugs for disease treatment. Because lipophilic agents can reside tightly into membranes, the encapsulation efficiency of lipophilic agents is always higher than the hydrophilic ones in liposomes. So far, numerous natural products have been investigated for the delivery systems using liposomes, such as Paclitaxel liposome (Li et al., 2024), liposomal resveratrol (Coimbra et al., 2012), vitamin A (Fan et al., 2023), and curcumin liposome (Wen et al., 2024). Ginsenoside Rg3 (Gs-Rg3) is a tetracyclic triterpenoid dammarane-type ginsenoside monomer extracted and purified from *Panax ginseng* C. A. Mey (P. ginseng). Gs-Rg3 possesses diverse biological activities, most notably neuroprotective effects. In the model mice of traumatic brain injury, Gs-Rg3 improved the neurological and motor functions of mice (Liu et al., 2024). In the cerebral ischemia-reperfusion injury rat model, Gs-Rg3 displayed an anti-mitochondrial oxidative stress effect by activating the Nrf2/HO-1 signaling pathway and promoting Nrf2 nuclear translocation, which significantly attenuated mitochondrial oxidative damage (Chen et al., 2024). In Alzheimer’s disease model, Gs-Rg3 ameliorated mitochondrial dysfunction, thereby alleviating cognitive impairments (Zhang et al., 2019).

Previous studies showed beneficial effcts of Gs-Rg3 on the symptoms of PD models. In rotenone-induced PD mice, Gs-Rg3 reduced the level of reactive oxygen species (ROS) and upregulated the expression of GCLM and GCLC (Han et al., 2021). In 6-OHDA-induced *Caenorhabditis elegans* model, it restored effectively the viability of dopaminergic neurons and extended the lifespan of *C. elegans*. Mechanistic studies indicated that Gs-Rg3 alleviated oxidative stress-caused damage in the nervous system of PD mice by activating the Nrf2/HO-1/NQO1 signaling pathway in the nigrostriatal system (Chalorak et al., 2021). The maintenance of cellular cardiolipin levels through controlling cardiolipin synthase is crucial for the prevention of PD. Gs-Rg3 augmented the interaction of growth factor receptor-bound protein 2 and tropomyosin-related kinase, modulating this pathway and elevating mitochondrial cardiolipin levels. Therefore, Gs-Rg3 exhibited promising potential for the prevention of PD by increasing cardiolipin expression and restoring mitochondrial homeostasis (Qi et al., 2024).

However, the therapeutic potential of Gs-Rg3 is constrained by its poor water solubility and low oral bioavailability. To address these limitations, Gs-Rg3 has been encapsulated within nano-systems such as liposomes, nanoparticles, and micelles to enhance its solubility and stability in aqueous solutions. Previous pharmacokinetic studies have reported that the ginsenoside Rg3 liposomes (Rg3-Lips) significantly enhanced the bioavailability of Gs-Rg3, with C_max_ and AUC values increased by 1.19-fold and 1.52-fold, respectively. Furthermore, the previous study reported that Rg3-Lips prolonged the circulation time of Gs-Rg3, with detectable plasma levels maintained at 12 h post-administration, whereas free Gs-Rg3 was nearly eliminated from circulation within 5 h Rg3-Lips enhanced the effects of Gs-Rg3 against A549 and HepG-2 cell lines and increased the antitumor efficacy of Gs-Rg3 in the tumor-bearing mice (Yu et al., 2013). This study aims to prepare Rg3-Lips and investigate their anti-PD effects using MPP^+^-induced SH-SY5Y cell and 1-methyl-4-phenyl-1,2,3,6-tetrahydropyridine (MPTP)-induced C57BL/6 mouse PD models.

## 2 Materials and methods

### 2.1 Materials

The following materials were used in the present study: ginsenoside Rg3 (Yuanye Biotechnology Co., Ltd., Purity ≥98%); soybean lecithin (Yuanye Biotechnology Co., Ltd.); cholesterol (Dibo Biotechnology Co., Ltd.); 1-methyl-4-phenylpyridinium (MPP^+^, Sigma-Aldrich); 1-methyl-4-phenyl-1,2,3,6-tetrahydropyridine (MPTP, Sigma-Aldrich); human neuroblastoma SH-SY5Y cell line (Punosai Life Technology Co., Ltd.); tyrosine hydroxylase (TH, Thermo Scientific Ltd.); Nrf2 antibody, Bax antibody, Bcl-2 antibody (Aimart Medical Technology Co., Ltd.); Cleaved Caspase-3 antibody (Cell Signaling Technology); GCLM (Abcam Ltd.); MEM/F12 medium, fetal bovine serum (FBS, Punosai Life Technology Co., Ltd.); lactate dehydrogenase (LDH), ROS, superoxide dismutase (SOD), glutathione (GSH) detection kits (Beyotime Biotechnology Co., Ltd.); TUNEL cell apoptosis detection kit (KeyGen Biotechnology Co., Ltd.); mitochondrial membrane potential detection kit (JC-1, Solarbio Science & Technology Co., Ltd.).

### 2.2 Preparation of Rg3-Lips

Rg3-Lips were prepared by the thin-film dispersion method. The formulation was optimized via single-factor experiments: Rg3-to-lipid mass ratio 1:15, phospholipid-to-cholesterol mass ratio 6:1, and ultrasonication time 4 min. An appropriate amount of Gs-Rg3, soy lecithin, and cholesterol was accurately weighed and dissolved in a mixed solvent of methanol and chloroform (4:1, v/v) via ultrasonication in a 37 °C water bath until fully dissolved. The organic solvent was removed by vacuum rotary evaporation at 40 °C to form a uniform lipid film on the inner wall of the flask. Sodium chloride solution was added to hydrate the film, and the particle size was reduced by probe ultrasonication. The resulting suspension was filtered through a 0.45 μm microporous membrane to obtain Rg3-Lips. The encapsulation efficiency was determined by ultrafiltration centrifugation. Briefly, 5 mL of Rg3-Lips solution was transferred to an ultrafiltration centrifuge tube and centrifuged at 3000 *g* for 30 min. The supernatant was collected and transferred to a 2 mL volumetric flask, followed by volume adjustment with methanol. The mass of free Gs-Rg3 (Wfree) was determined by HPLC using an Agilent 5 TC-C18 column (4.6 × 250 mm, 5 μm). The mobile phase consisted of acetonitrile and water (58:42, v/v), with a flow rate of 1.0 mL/min. The column temperature was maintained at 25 °C, the detection wavelength was 203 nm, and the injection volume was 20 μL. Separately, 1 mL of Rg3-Lips solution was placed in a 5 mL volumetric flask, made up to volume with methanol, and vortexed to break the emulsion. After filtration through a 0.45 μm microporous membrane, the total mass of Gs-Rg3 (W_total_) was measured by HPLC. The encapsulation efficiency was calculated using the formula: Encapsulation efficiency (%) = (1 - W_free_/W_total_) × 100%.

### 2.3 Characterization of Rg3-Lips

A 1 mL aliquot of Rg3-Lips solution was transferred to a nanoparticle size and Zeta potential analyzer, equilibrated at 25 °C for 60 s, and then particle size and Zeta potential were measured. The liposome sample was diluted appropriately, applied to a carbon-coated copper grid, and stained with 2% phosphotungstic acid (w/v) using the negative staining method. After air-drying at room temperature, the morphology of Rg3-Lips was observed by transmission electron microscopy (TEM). Rg3-Lips was sealed in a dialysis bag and immersed in pH 7.4 PBS buffer containing 2% Tween 80, followed by incubation at 37 °C with constant stirring at 120 r/min. Samples were collected at 0.5, 1, 2, 4, 8, 12, 24, and 48 h, and an equal volume of fresh blank dialysis buffer was replenished immediately after each sampling. The experiment was performed in triplicate. The Gs-Rg3 contents in the dialysate was determined by HPLC, and the cumulative release rate was calculated.

### 2.4 *In vitro* experiments

#### 2.4.1 Experimental design

SH-SY5Y cells were cultured in MEM/F12 medium supplemented with 15% fetal bovine serum and 1% penicillin-streptomycin solution. Subsequently, they were treated with 2 mM MPP^+^ for different durations: 2, 4, 6, 12, 24, and 48 h. To investigate the safety of Rg3-Lips, SH-SY5Y cells were treated with Rg3-Lips at concentrations ranging from 0 to 40 μM. Based on initial screening (0–40 μM), SH-SY5Y cells were pre-treated with Rg3-Lips (0.03125, 0.0625, or 0.125 μM) for 6 h prior to a 24 h challenge with 2 mM MPP^+^.

#### 2.4.2 Cell viability

The cell survival rate was quantified using an MTT assay, with absorbance measurements taken at 490 nm (Ma et al., 2025).

#### 2.4.3 LDH assay

LDH release reagent (10 µL) was added to each well, and the plate was incubated for 1 h. The supernatant from each well was collected and centrifuged at 1500 *g* for 5 min. A 120 µL aliquot of the supernatant was combined with 60 µL of LDH detection working solution. The mixture was incubated in the dark on a shaker at room temperature for 30 min. Absorbance was measured at 490 nm, and LDH levels were calculated.

#### 2.4.4 ROS levels

The cultivated cells were incubated in MEM/F12 medium containing DCFH-DA at 37 °C for 30 min. The medium was removed and the cells were washed with serum-free medium for 3 times. Fluorescence intensity was assayed using a laser confocal microscope by experimenters who were blinded to the groups. For quantitative analysis, DCFH-DA fluorescence images were analyzed using ImageJ software. Equal-sized regions were selected in each field of view, and fluorescence intensity was measured. The mean fluorescence intensity of the control group served as the reference. Data were normalized and expressed as fold change relative to the control.

#### 2.4.5 Mitochondrial membrane potential

The culture medium was removed and SH-SY5Y cells were washed with serum-free medium for 1 time. Added 1 mL of serum-free culture medium with 1 mL of JC-1 staining working solution, mixed, and incubated for 20 min. Discarded the supernatant. Washed the cells twice with 1 mL of 1× JC-1 staining buffer per wash. Add 1 mL of fresh culture medium. Visualize and quantify mitochondrial membrane potential by measuring red and green fluorescence using a laser confocal microscope by experimenters who were blinded to the groups.

#### 2.4.6 Assay of SOD and GSH

Cells were digested with 0.25% trypsin and centrifuged at 1000 *g* for 10 min. After discarding the supernatant, the cell pellet was washed once with 1 mL of PBS. The pellet was then resuspended in 0.5 mL of PBS and lysed by sonication. Following another centrifugation at 1000 *g* for 10 min, the supernatant was collected. The total protein concentration was determined using a BCA assay kit. SOD activity and GSH levels were measured according to the manufacturer’s instructions.

#### 2.4.7 TUNEL staining

The cells were fixed with 4% paraformaldehyde, washed with PBS, and permeabilized with 1% Triton X-100 at room temperature. After PBS washes, 100 µL of Streptavidin-FITC working solution was added, and the cells were incubated at 37 °C in the dark for 2 h. Following three PBS washes, the nuclei were stained with DAPI in the dark at room temperature. The images were acquired using a laser confocal microscope by experimenters who were blinded to the groups. For quantitative analysis, the numbers of DAPI-positive cells and TUNEL-positive cells were counted within defined fields of view using ImageJ software. The apoptotic rate was calculated as TUNEL-positive cells divided by DAPI-positive cells × 100%.

#### 2.4.8 Immunofluorescence

The cells were fixed with 4% paraformaldehyde. After permeabilization with 1% Triton X-100 at room temperature for 15 min, the cells were washed with PBS and blocked with blocking solution for 1 h. The blocking solution was then replaced with a solution of Nrf2 primary antibody (1:300), and cells were incubated overnight at 4 °C. The following day, the primary antibody was removed, and cells were washed 3 times with PBS (5 min per wash). Cells were then incubated with an Alexa Fluor 488-conjugated goat anti-rabbit IgG secondary antibody (1:400) for 2 h at room temperature in the dark. After 3 additional PBS washes (5 min per wash), cell nuclei were stained with DAPI in the dark for 10 min at room temperature. Finally, the DAPI solution was removed, and cells were observed and imaged using a laser confocal microscope by experimenters who were blinded to the groups.

#### 2.4.9 Western blot

Cells were lysed on ice for 30 min using RIPA lysis buffer. The lysates were centrifuged at 12000 *g* for 15 min, and the supernatant was collected. The total protein concentration was determined using a BCA assay kit. For nuclear-cytoplasmic fractionation, cells were processed with extraction reagents following the manufacturer’s protocol. Protein samples were mixed with 4× loading buffer, denatured by boiling, and stored at -20 °C. Proteins were separated by SDS-PAGE. Electrophoresis was performed initially at 80 V for 30 min, followed by 120 V until the electrophoresis is completed. The separated proteins were then transferred onto a PVDF membrane. The membrane was blocked with a blocking solution for 15 min at room temperature with gentle shaking and subsequently washed 3 times with TBST (5 min per wash). It was then incubated with the primary antibody overnight at 4 °C. After washing with TBST, the membrane was incubated with a horseradish peroxidase-conjugated secondary antibody for 1 h at room temperature. Following final TBST washes, protein bands were visualized using an enhanced chemiluminescence substrate, and band intensities were quantified using ImageJ software.

### 2.5 *In vivo* experiments

#### 2.5.1 Experimental design

Male C57BL/6 mice (18–25 g) were obtained from Jinan Pengyue Laboratory Animal Breeding Co., Ltd (license number: SCXK (Lu) 20220006). Animals were acclimatized for 3 days before experimentation under the following housing conditions: temperature of 22 °C ± 2 °C, relative humidity of 50%, and a 12 h light/12 h dark cycle. Food and water were provided *ad libitum* throughout. The mice were randomly divided into 4 groups (n = 15 per group): control, model, Gs-Rg3, and Rg3-Lips, according to a random allocation procedure. From Day 1 to Day 7, mice in the Gs-Rg3 group received Gs-Rg3 (2.5 mg/kg) by gavage, while mice in the Rg3-Lips group received Rg3-Lips (2.5 mg/kg) by intraperitoneal injection. From Day 8 to Day 12, the corresponding Gs-Rg3 or Rg3-Lips treatment was continued once daily, and MPTP (30 mg/kg) was intraperitoneally administered 2 h after each treatment. Behavioral tests were performed on Days 13 and 14 after the final MPTP administration. The open field test was performed on Day 13, followed by the pole test and rotarod test on Day 14. The testing order was kept consistent across all groups to minimize potential carry-over effects.

#### 2.5.2 Pole test

This test was performed to assess the balance and motor coordination of mice. Briefly, each mouse was gently caught by the tail and placed atop the ball on the pole. Once the hind paws securely gripped the ball, the mouse was released to initiate the trial. The latency for the mouse to descend the pole and land with all four limbs on the base was recorded. Each animal was tested three times with a 5 min inter-trial interval (Yang L. et al., 2025). Two mice in each group were excluded because they either took too long to climb the pole (exceeding 60 s) or refused to climb. Consequently, the subsequent experiments were conducted with 13 animals per group.

#### 2.5.3 Rotarod test

The rotarod test was conducted. One mouse from each of the four experimental groups was randomly assigned to a chamber per run. Each trial began with a 3 min adaptation phase at a constant speed of 5 rpm. The speed was then progressively increased, and the latency to fall was recorded for each mouse. Following a fall, the mouse was returned to its home cage for a 30 min rest before the next trial. Each mouse completed three trials, and the average latency across trials was calculated for analysis (Mohammed et al., 2024).

#### 2.5.4 Open field test

Each mouse was placed in the center of a 50 × 50 cm arena and allowed 3 min for habituation. Subsequently, activity was recorded for 5 min. Testing occurred in a quiet, dimly lit room to reduce stress and external stimuli. After each trial, the arena was cleaned with 75% ethanol to prevent odor-based interference. The motion trajectory was recorded with a camera and analyzed with a Smart Video Tracking Software 3.0 (Panlab, S.L., Barcelona, Spain) (Zhu et al., 2025).

#### 2.5.5 Immunohistochemistry

Three mice per group were anesthetized with isoflurane. The brain was harvested and immersed in 4% paraformaldehyde solution for 24 h. The tissue was then dehydrated through a graded ethanol series, cleared in xylene (twice, 5 min each), and embedded in paraffin. Sections were deparaffinized, rehydrated, and washed three times in PBS (3 min per wash). Endogenous peroxidase activity was quenched with a 10-min incubation in 3% hydrogen peroxide. After washing, sections were blocked with 10% normal goat serum for 20 min. For immunohistochemical staining, sections were incubated overnight at 4 °C in a humidified chamber with a primary antibody against TH (1:200). After washing, sections were incubated with a biotin-labeled goat anti-rabbit IgG secondary antibody for 60 min at 37 °C. Following another wash, the signal was amplified by incubation with streptavidin-peroxidase for 60 min at 37 °C. Coloration was performed using DAB substrate for 5 min, and then stained sections were imaged under a light microscope. For quantitative analysis, TH-positive neurons were determined in 5 sections of the substantia nigra corresponding to an area from 2.90 mm to 3.68 mm from the Bregma in mice. A 150 × 150 μm^2^ grid was randomly placed on each section of the image. The tyrosine hydroxylase-positive nerve fibers in the striatum were quantified using Image J software.

#### 2.5.6 TUNEL staining

Three mice per group were used for TUNEL staining, which was performed according to a previously reported protocol.

#### 2.5.7 Assay of GSH

Five mice per group were used for GSH analysis. Mice were anesthetized with isoflurane, and brains were rapidly dissected and weighed. Tissue was homogenized using a tissue homogenizer, and the lysate was centrifuged at 2500 *g* for 10 min. The supernatant was used for subsequent analysis: total protein concentration was measured, and GSH levels were assessed following the manufacturer’s instruction.

#### 2.5.8 Western blot

Western blot was conducted according to previous protocol. The concentrations of primary antibodies: rabbit anti-Nrf2 (1:1000), rabbit anti-GCLM (1:1000), rabbit anti-Bax (1:1000), rabbit anti-Bcl-2 (1:1000), rabbit anti-Cleaved caspase-3 (1:1000), and rabbit anti-GAPDH (1:1000).

### 2.6 Statistical analysis

Statistical analyses were performed using GraphPad Prism 9.0 software. The data were presented as mean ± standard deviation (SD). The normality of data was assessed using the Shapiro-Wilk test. For data meeting normal distribution, one-way ANOVA followed by Tukey’s *post hoc* test was applied. Otherwise, the nonparametric Kruskal–Wallis test with Dunn’s *post hoc* test was used. A P value <0.05 was considered statistically significant.

## 3 Results

### 3.1 Preparation and characterization of Rg3-Lips

The encapsulation efficiency (EE) of Rg3-Lips was investigated. EE showed an initial increase, then decreased as the lecithin-to-Gs-Rg3 ratio rose. The optimal lecithin-to-Gs-Rg3 ratio was 15:1, yielding the highest EE of 95.68% (Figure 1A). The lecithin-to-cholesterol ratio was then investigated while maintaining a constant lecithin-to-Gs-Rg3 ratio of 15:1. The optimal ratio was found to be 6:1, resulting in a maximum EE of 92.10% (Figure 1B). Ultrasound time was optimized using the established ratios (15:1 lecithin-Gs-Rg3, 6:1 lecithin-cholesterol). EE peaked at 97.16% after 4 min of ultrasound (Figure 1C). Consequently, a lecithin-to-Gs-Rg3 ratio of 15:1, a lecithin-to-cholesterol ratio of 6:1, and an ultrasound time of 4 min were identified as the optimal conditions for preparing Rg3-Lips.

**FIGURE 1:**
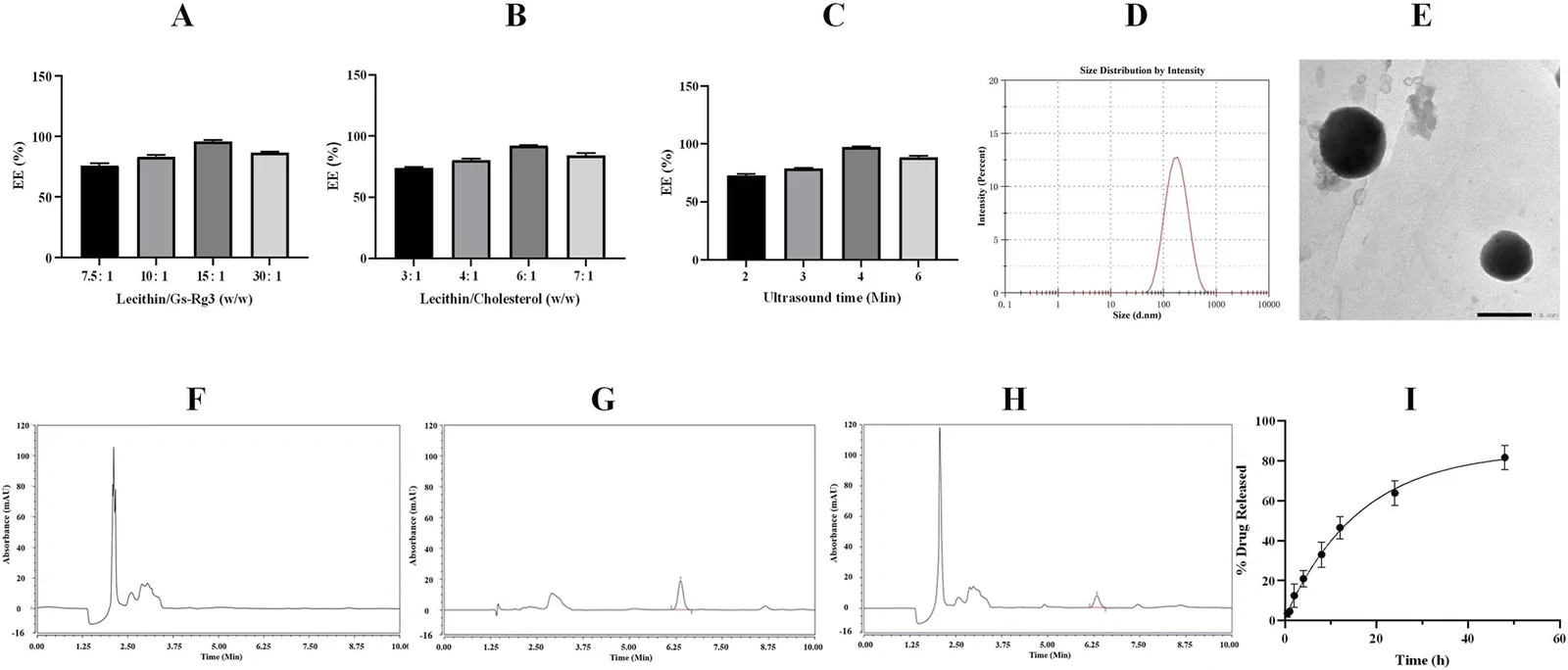
Preparation and characterization of Rg3-Lips. **(A)** Lecithin/Gs-Rg3; **(B)** Lecithin/cholesterol; **(C)** Ultrasound time; **(D)** Particle size distribution of Rg3-Lips; **(E)** Representative morphology of Rg3-Lips; **(F)** Blank liposomes; **(G)** Gs-Rg3; **(H)** Rg3-Lips; **(I)** *In vitro* release of Rg3-Lips. Data are presented as mean ± SD (n = 3).

The reliability of the optimized process was verified through three independent batches of Rg3-Lips, which demonstrated consistent EE (Table 1). The optimized Rg3-Lips formulation had an average particle size of 170.78 nm and a PDI of 0.261, confirming a monodisperse distribution (PDI <0.3) (Figure 1D). A zeta potential of −1.92 mV suggested adequate electrostatic stabilization for colloidal stability. Prepared under the optimal protocol, the formulation yielded a high EE (93.33%) and provided sustained release, with 81.6% of the drug released cumulatively within 48 h *in vitro*. TEM analysis revealed the formation of spherical liposomes possessing a clear bilayer structure, with sizes ranging from 100 to 200 nm, in agreement with dynamic light scattering measurements (Figure 1E).

**TABLE 1:** Encapsulation efficiency of Rg3-Lips.

Samples	EE (%)	Average EE (%)	RSD (%)
1	91.90	​	​
2	92.94	93.33	1.77
3	95.14	​	​

The results showed that Gs-Rg3 was well separated from matrix components, evidenced by sharp chromatographic peaks and no interference detected in the negative control, confirming the high specificity of the method (Figures 1F– H). In the linearity study, the peak area (Y) of Gs-Rg3 was plotted versus its concentration (X, μg/mL), yielding the standard curve equation Y = 0.07X + 0.2395 (R^2^ = 0.9992). This finding confirms excellent linearity over the concentration range of 2–200 μg/mL. For precision assessment, replicate injections of Gs-Rg3 standard solutions at 10, 40, and 80 μg/mL were analyzed, yielding relative standard deviation (RSD) values of 1.01%, 0.31%, and 0.28%, respectively. All RSDs were below the 2% which was considered as acceptance threshold, indicating a good precision of the HPLC system (Table 2). To evaluate solution stability, aliquots of the test solution were analyzed at 0, 2, 4, 6, 12, and 24 h post-preparation. The resulting RSD of 1.88% (RSD <2%) confirmed that the test solution remained stable over 24-h period (Table 3). In spiked recovery experiments, the recovery rates of Gs-Rg3 were in the range of 98.50%–108.60%, with RSD values below 2% across all concentration levels. These results demonstrate that the analytical method is accurate, reliable, and suitable for the quantitative determination of Gs-Rg3 (Table 4).

**TABLE 2:** Relative standard deviation.

Doses (μg/mL)	Samples						RSD (%)
	1	2	3	4	5	6	
10	0.9043	0.8847	0.8813	0.8800	0.8833	0.8846	1.01
40	3.1202	3.1446	3.1363	3.1381	3.1414	3.1482	0.31
80	6.0062	6.0420	6.0220	5.9946	6.0180	6.0036	0.28

**TABLE 3:** Stability evaluation.

Time (h)	Peak area	RSD (%)
0	1.3017	​
2	1.3029	​
4	1.3376	1.88
6	1.3202	​
12	1.3424	​
24	1.3658	​

**TABLE 4:** Sample recovery.

TC (μg/mL)	MC (μg/mL)	Recovery (%)	RSD (%)
10.00	10.11 9.97 10.36	101.10 99.70 108.60	1.95
40.00	41.07 40.76 39.80	102.68 101.90 99.50	1.63
60.00	61.20 59.10 60.98	102.00 98.50 101.63	1.91

For the *in vitro* release profile of Rg3-Lips, rapid cumulative release of Gs-Rg3 was observed within the first 12 h, followed by a gradual decline in the release rate thereafter. By 48 h, the cumulative release of Gs-Rg3 from Rg3-Lips had reached 81.6% (Figure 1I).

### 3.2 Effect of Rg3-Lips on MPP^+^-induced injury of SH-SY5Y cells

SH-SY5Y cells were exposed with 2 mM MPP^+^ for 2, 6, 12, 24, and 48 h. MPP^+^ exposure significantly reduced survival in a time-dependent manner, with notable decreases observed at 12, 24, and 48 h compared to the control group (P < 0.01). Based on these results, a 24-h MPP^+^ exposure was selected as the optimal condition for establishing the cellular injury model in subsequent experiments (Figure 2A). Rg3-Lips exposure for 24 h showed no significant effect on SH-SY5Y cell survival at concentrations up to 10 μM, whereas concentrations of 20 μM or higher caused a marked reduction in survival (P < 0.01), indicating cytotoxic effects of Rg3-Lips. These findings suggested the concentration range of 0–10 μM was the safe dosage range for Rg3-Lips (Figure 2B). Compared to the control, cell viability was significantly lower in the model group (P < 0.01). Treatment with Rg3-Lips (0.03125–0.125 μM) significantly restored viability in a dose-dependent manner (P < 0.05 or P < 0.01) (Figure 2C). Additionally, LDH release increased significantly in the model group compared to the control (P < 0.01). Rg3-Lips treatment reduced the LDH release (P < 0.01) (Figure 2D).

**FIGURE 2:**
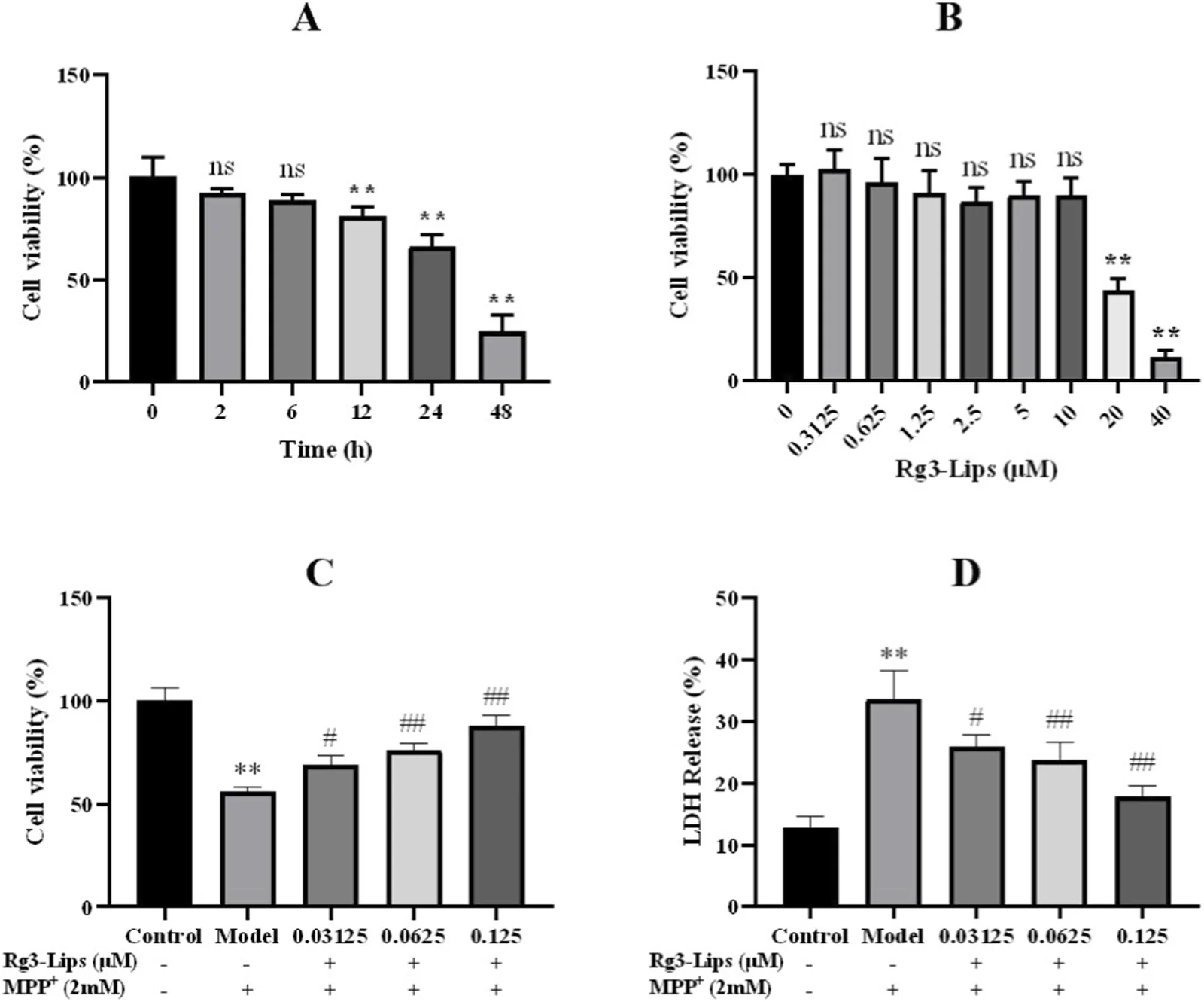
Effect of Rg3-Lips on MPP^+^-induced injury of SH-SY5Y cells. **(A)** MPP^+^ exposure; **(B)** Rg3-Lips exposure; **(C)** Effect of Rg3-Lips on cell viability of SH-SY5Y cells exposed to MPP^+^; **(D)** Effect of Rg3-Lips on LDH release. Data are presented as mean ± SD (n = 3). Statistical analysis was performed using one-way ANOVA followed by Tukey’s *post hoc* test. **P < 0.01, compared with the control group; ^#^P < 0.05, ^##^P < 0.01, compared with the model group.

### 3.3 Effect of Rg3-lips on oxidative stress in SH-SY5Y cells

Total intracellular ROS in SH-SY5Y cells were measured using the DCFH-DA probe, which produces a green fluorescence signal proportional to ROS levels (Figure 3A). Compared with the control group, MPP^+^ exposure significantly enhanced ROS levels (P < 0.01, Figure 3B). In contrast, Rg3-Lips at concentration of 0.0625 and 0.125 μM markedly reduced ROS levels compared with the model group (P < 0.01). As shown in Figures 3C,D, compared with the control group, SOD activity and GSH levels in the model group decreased significantly (P < 0.01). While Rg3-Lips treatment increased SOD activity and GSH levels (P < 0.05 or P < 0.01), indicating that Rg3-Lips enhanced the antioxidant capacity and then scavenged ROS. Compared with the model group, Rg3-Lips enhanced Nrf2 nuclear translocation in SH-SY5Y cells (Figure 3E). Compared with the control group, the model group exhibited significantly reduced expression of nuclear Nrf2 and GCLM proteins (P < 0.05 or P < 0.01). While Rg3-Lips increased the levels of nuclear Nrf2 and GCLM as compared to the model group (P < 0.05 or P < 0.01) (Figures 3F,G).

**FIGURE 3:**
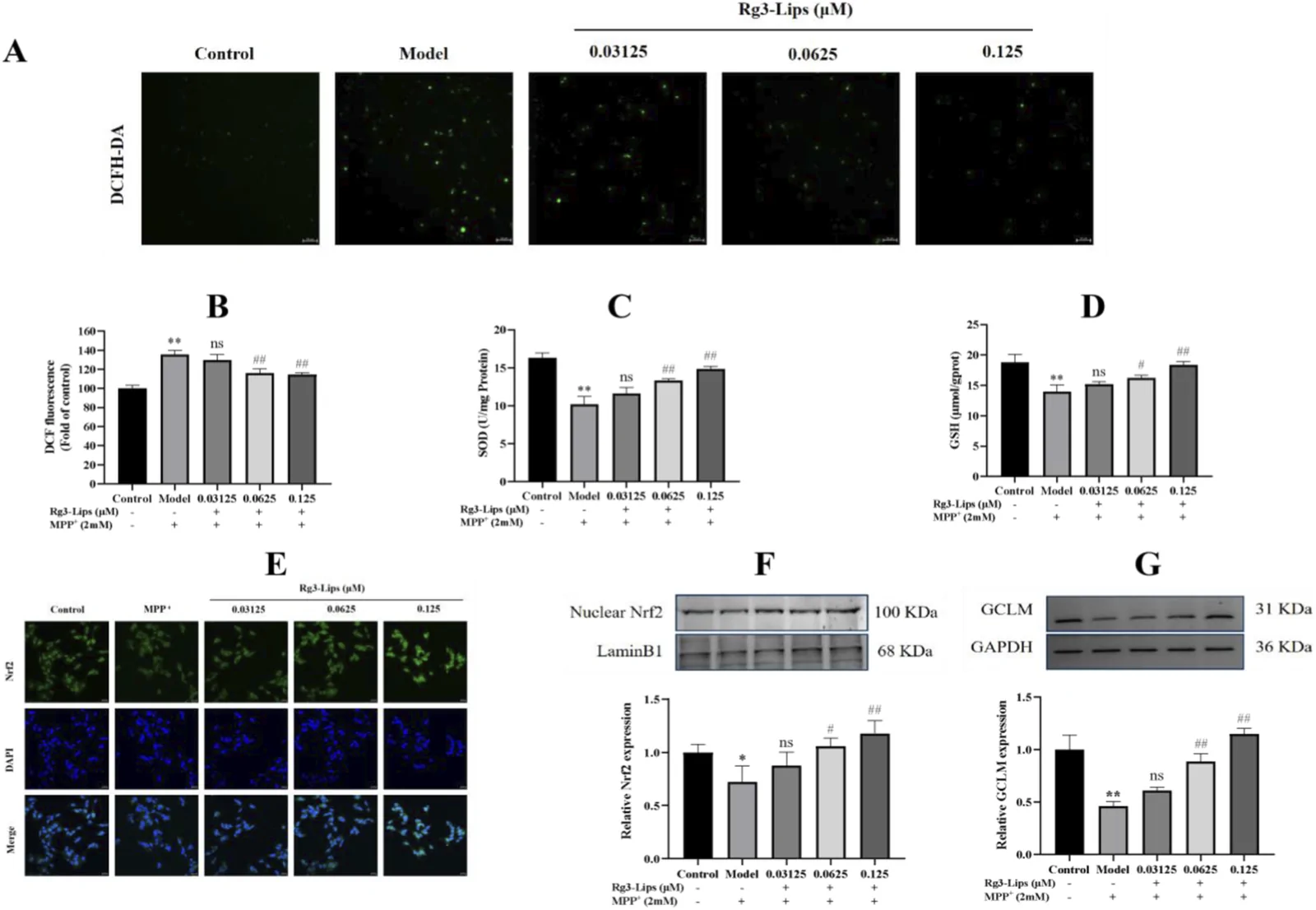
Effect of Rg3-Lips on oxidative stress in SH-SY5Y cells. **(A)** ROS; **(B)** Quantification of ROS; DCFH-DA fluorescence intensity was quantified using ImageJ in regions of the same size in each field of view and normalized to the mean fluorescence intensity of the control group. **(C)** SOD activity; **(D)** GSH levels; **(E)** Representative pictures of Nrf2 nuclear translocation; **(F)** Representative pictures and bar graphs of quantitative analysis of nuclear Nrf2 in Western blot; **(G)** Representative pictures and bar graphs of quantitative analysis of GCLM in Western blot. Data are presented as mean ± SD (n = 3). Statistical analysis was performed using one-way ANOVA followed by Tukey’s *post hoc* test. **P < 0.01, compared with the control group; ^#^P < 0.05, ^##^P < 0.01, compared with the model group.

### 3.4 Effect of Rg3-lips on apoptosis in SH-SY5Y cells

A loss of mitochondrial membrane potential is a key early indicator of mitochondrial dysfunction and the initiation of the intrinsic apoptotic pathway. Figure 4A and B showed that the model group exhibited a significant increase in green fluorescence intensity and a decrease in red fluorescence intensity compared with the control group (P < 0.01), which indicated that the mitochondrial membrane potential of model group significantly reduced. However, Rg3-Lips not only elevated the red fluorescence intensity but also reduced the green fluorescence intensity, demonstrating the mitochondrial membrane potential of Rg3-Lips group increased compared with the model group (P < 0.05). In Figures 4C,D, the number of TUNEL-positive cells was significantly higher in the model group than the control group (P < 0.01). Compared to the model group, treatment with Rg3-Lips at concentration of 0.0625 and 0.125 μM significantly reduced the number of TUNEL-positive cells (P < 0.01). The Bcl-2/Bax ratio is a key regulatory switch controlling mitochondrial apoptosis initiation. A decreased ratio increases the level of Cleaved Caspase-3 which is the marker of irreversible execution-phase apoptosis. In the model group, the Bcl-2/Bax ratio significantly decreased (P < 0.01) and the Cleved Caspase-3 contents augmented (P < 0.01). Compared with the model group, Rg3-Lips not only enhanced Bcl-2/Bax ratio but also reduced Cleaved Caspase-3 contents (P < 0.01) (Figures 3F,G).

**FIGURE 4:**
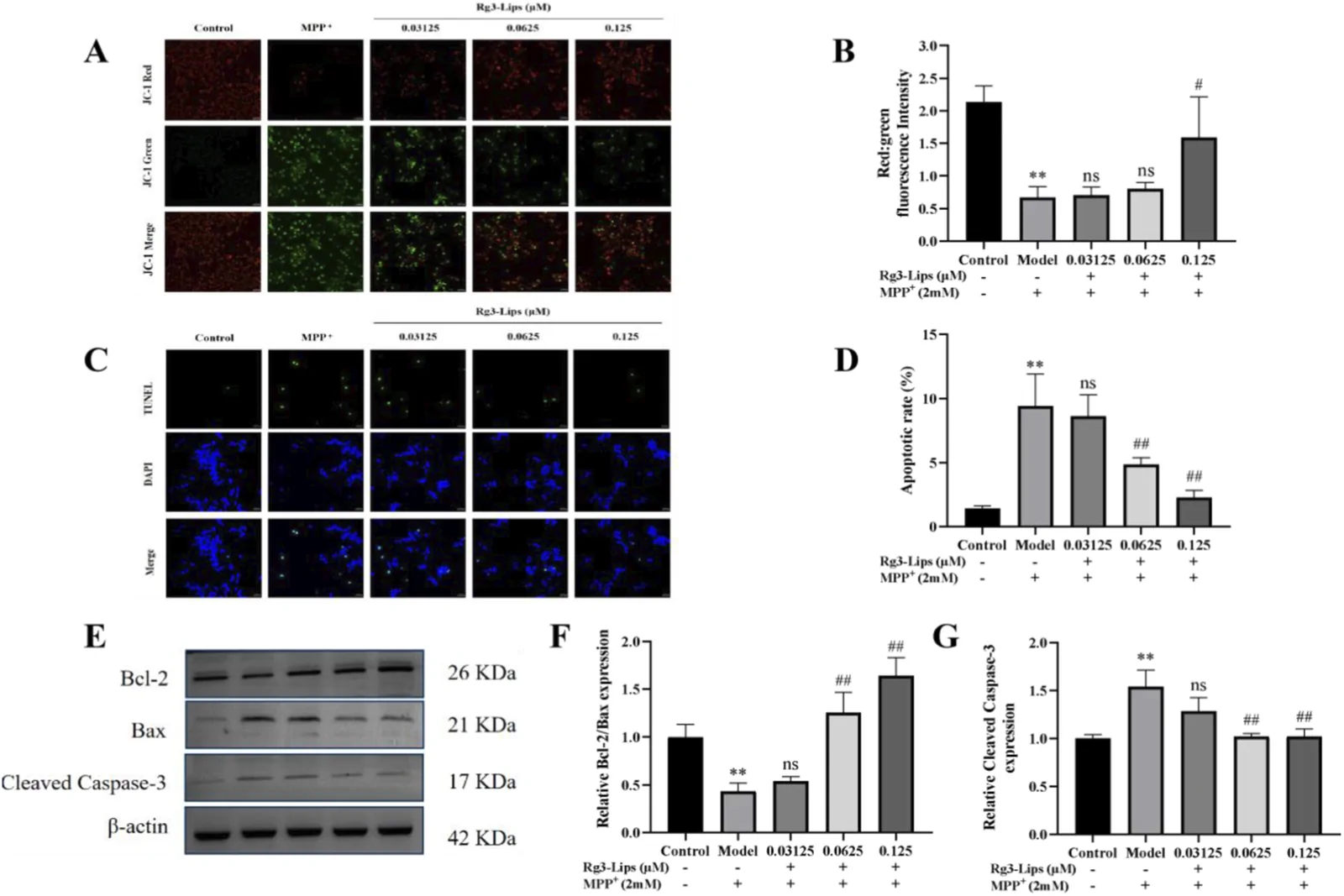
Effect of Rg3-Lips on the mitochondrial membrane potential and apoptosis in SH-SY5Y cells. **(A)** Representative pictures of JC-1 staining; **(B)** Bar graphs of quantitative analysis of the JC-1 red/green fluorescence ratio; **(C)** TUNEL staining; **(D)** Bar graphs of quantitative analysis of TUNEL-positive cells; The numbers of DAPI-positive cells and TUNEL-positive cells were counted within defined fields of view using ImageJ software, and the apoptotic rate was calculated as the number of TUNEL-positive cells divided by the total number of DAPI-positive cells × 100%. **(E)** Representative pictures of Bcl-2, Bax, Cleaved Caspase-3 in Western blot; **(F)** Bar graphs of quantitative analysis of Bcl-2/Bax; **(G)** Bar graphs of quantitative analysis of Cleaved Caspase-3. **P < 0.01, compared with the control group; Data are presented as mean ± SD (n = 3). Statistical analysis was performed using one-way ANOVA followed by Tukey’s *post hoc* test. ^#^P < 0.05, ^##^P < 0.01, compared with the model group.

### 3.5 Effect of Rg3-Lips on motor functions of PD mice

The model group exhibited a markedly prolonged climbing time compared to the control group (P < 0.01). Conversely, the climbing time was significantly shortened in the Rg3-Lips group (P < 0.01). And the climbing time of Gs-Rg3 group did not display a significant decrease as compared with the model group (Figure 5A). In line with the findings of pole test, the rotarod retention time was significantly shortened in the model group compared to control (P < 0.01). Rg3-Lips increased the rotarod retention time (P < 0.01). While the rotarod retention time of Gs-Rg3 group did not show significant changes as compared to the model group (Figure 5B). Compared with the control group, the total distance and mean speed of mice in the model group decreased (P < 0.01). Rg3-Lips group exhibited a significant increase in both total distance and mean speed (P < 0.05). Treatment with Gs-Rg3 did not elevate significantly total distance and mean speed (Figures 5C–E).

**FIGURE 5:**
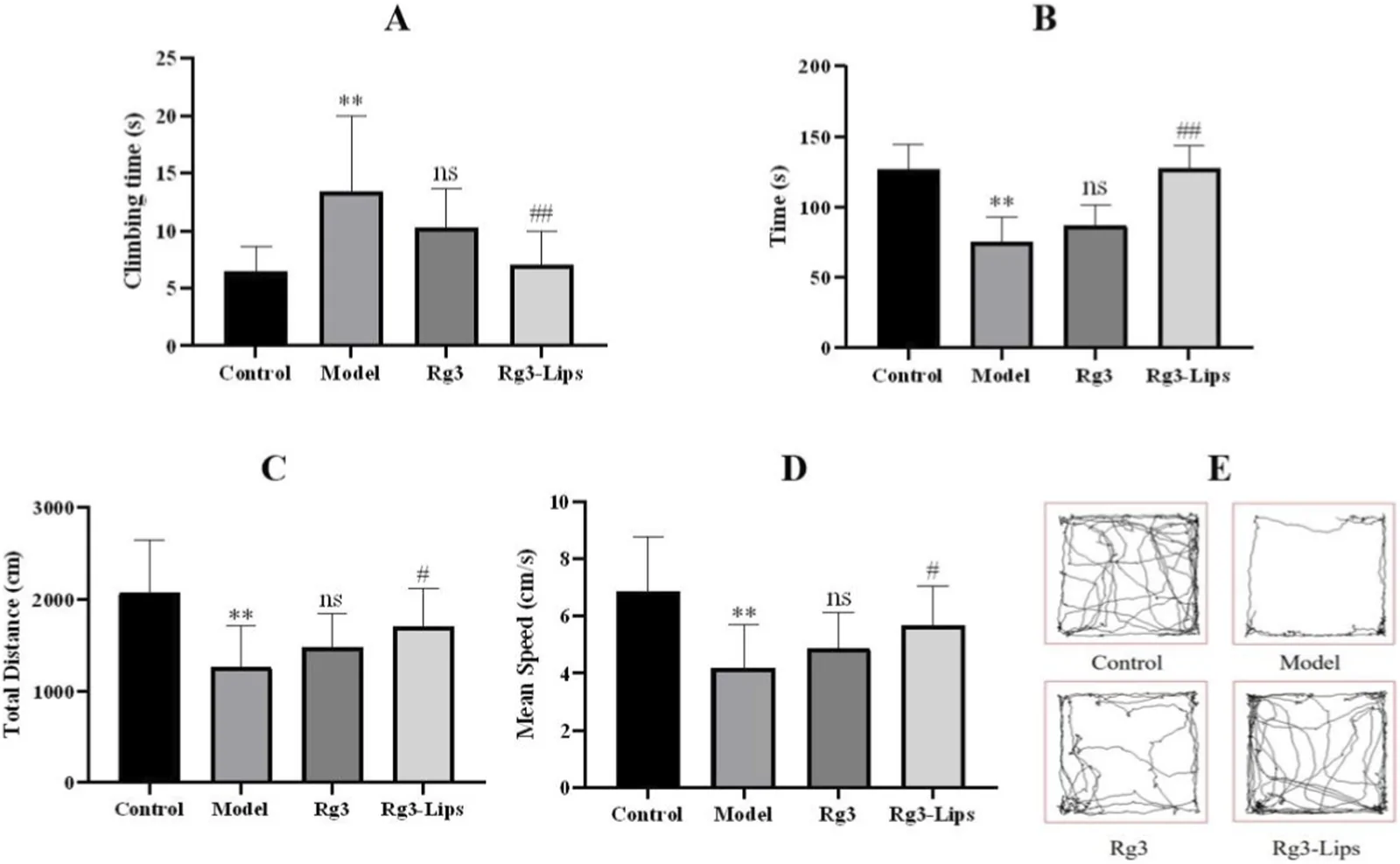
Effect of Rg3-Lips on motor functions of PD mice. **(A)** Climbing time; **(B)** Rotarod retention time; **(C)** Total distance; **(D)** Mean speed; E, Representative pictures of trajectory of mice. Data are presented as mean ± SD (n = 13). A and B were analyzed using the Kruskal–Wallis test followed by Dunn’s *post hoc* test, while C and D were analyzed using one-way ANOVA followed by Tukey’s *post hoc* test. **P < 0.01, compared with the control group; ^#^P < 0.05, compared with the model group.

### 3.6 Effect of Rg3-Lips on dopaminergic nerve of PD mice

Compared with the control group, the number of tyrosine hydroxylase (TH)-positive neurons in the substantia nigra and density of TH-positive nerve fibers in the striatum of mice in model group significantly reduced (P < 0.01). Compared with the model group, Rg3-Lips administration augmented the number of TH-positive neurons in the substantia nigra and density of TH-positive nerve fibers in the striatum (P < 0.01). However, Gs-Rg3 did not display a significant attenuation of injury of dopaminergic nerve in PD mice (Figure 6).

**FIGURE 6:**
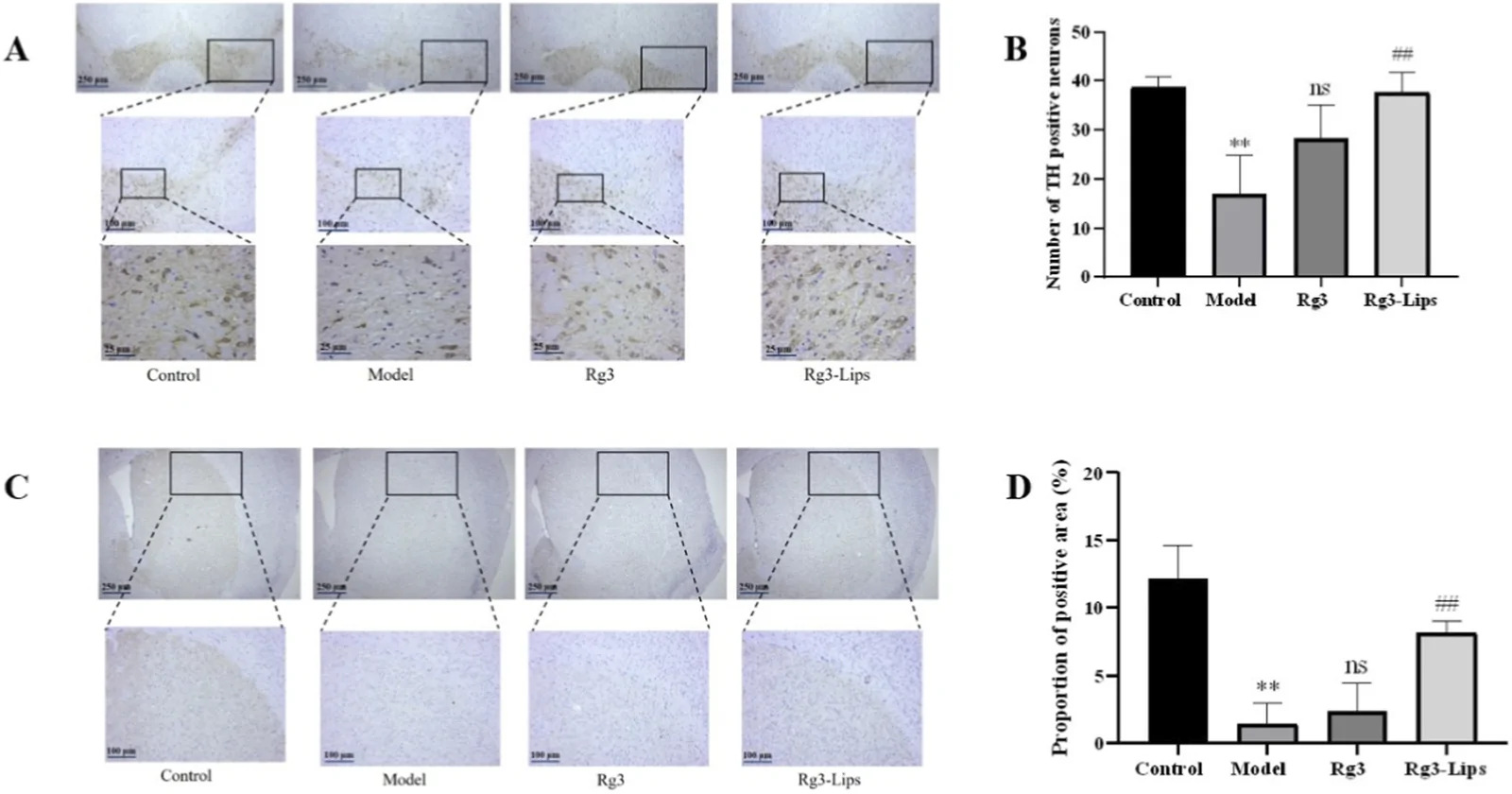
Effect of Rg3-Lips on dopaminergic nerve of PD mice. **(A)** TH staining of substantia nigra; **(B)** Bar graphs of quantitative analysis of TH-positive cells; **(C)** TH staining of striatum; **(D)** Bar graphs of quantitative analysis of TH-positive fibers. Data are presented as mean ± SD (n = 3). Statistical analysis was performed using one-way ANOVA followed by Tukey’s *post hoc* test. **P < 0.01, compared with the control group; ^##^P < 0.01, compared with the model group.

### 3.7 Effect of Rg3-Lips on oxidative stress in PD mice

Compared with the control group, the contents of nuclear Nrf2 and GCLM in the model group were significantly decreased (P < 0.05 or P < 0.01). Compared with the model group, Rg3-Lips enhanced the levels of Nrf2 and GCLM (P < 0.05). Compared with the control group, the GSH levels in the substantia nigra and striatum of mice in the model group were significantly reduced (P < 0.05 or P < 0.01). In contrast, the Rg3-Lips group exhibited a significant elevation in GSH levels (P < 0.05 or P < 0.01) (Figure 7). However, the Gs-Rg3 group did not show significant changes in nuclear Nrf2, GCLM and GSH.

**FIGURE 7:**
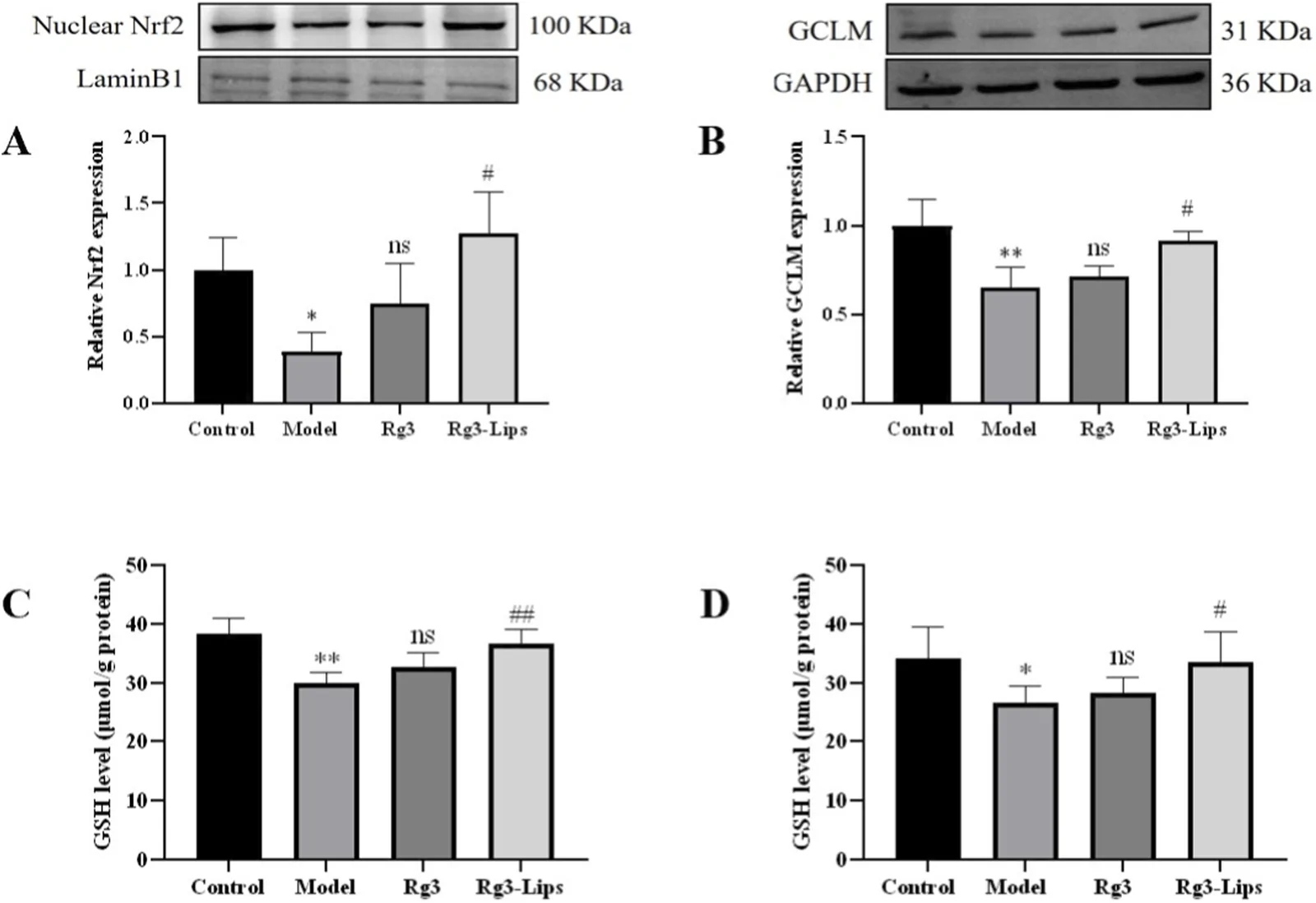
Effect of Rg3-Lips on oxidative stress in PD mice. **(A)** Nuclear Nrf2 (n = 3); **(B)** GCLM (n = 3); **(C)** GSH in the substantia nigra of mice (n = 5); **(D)** GSH in the striatum of mice (n = 5); Data are presented as mean ± SD. **(A,C,D)** were analyzed using one-way ANOVA followed by Tukey’s *post hoc* test, while **(B)** was analyzed using the Kruskal–Wallis test followed by Dunn’s *post hoc* test. *P < 0.05, **P < 0.01, compared with the control group; ^#^P < 0.05, ^##^P < 0.01, compared with the model group.

### 3.8 Effect of Rg3-Lips on apopotosis in PD mice

Compared with the control group, the number of apoptotic cells in the substantia nigra of mice in the model group was significantly increased (P < 0.01). Compared with the model group, the Rg3-Lips group displayed a significant decrease in the number of apoptotic cells in the substantia nigra (P < 0.01) (Figures 8A,B). Compared with the control group, the model group showed a decrease of Bcl-2/Bax ratio (P < 0.01) and an increase of Cleaved Caspase-3 contents (P < 0.01). Rg3-Lips group exhibited a significant increase of Bcl-2/Bax ratio (P < 0.01) and reduction of Cleaved Caspase-3 levels (P < 0.01) as compared to the model group (Figures 8C–E). Compared to the model group, Gs-Rg3 administration did not cause significant changes in the number of apoptotic cells, Bcl-2/Bax ratio, and Cleaved Caspase-3 contents.

**FIGURE 8:**
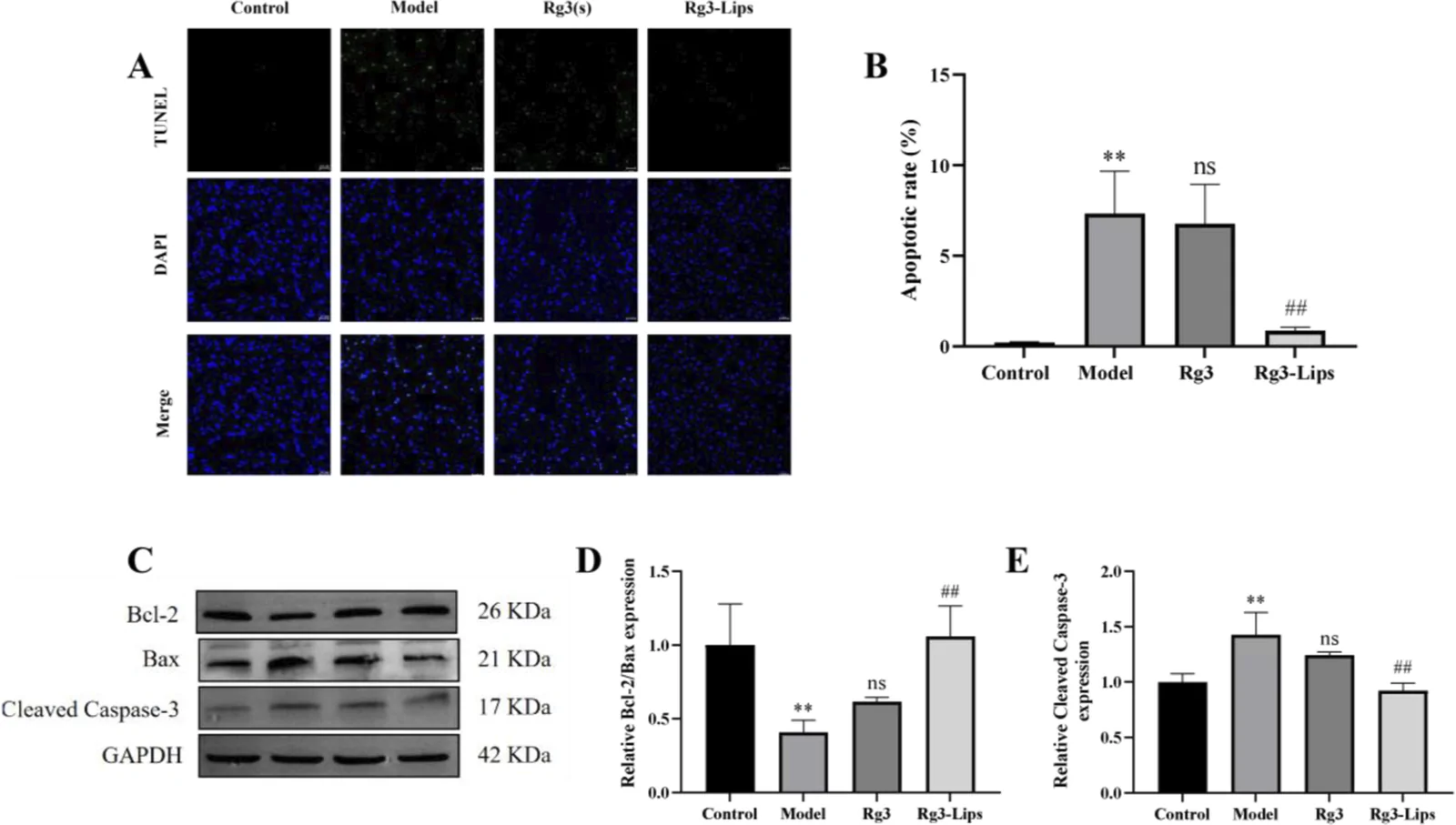
Effect of Rg3-Lips on apopotosis in PD mice. **(A)** TUNEL staining; **(B)** Bar graphs of quantitative analysis of TUNEL-positive cells; **(C)** Representative pictures of Bcl-2, Bax, Cleaved Caspase-3 in Western blot; **(D)** Bar graphs of quantitative analysis of Bcl-2/Bax; **(E)** Bar graphs of quantitative analysis of Cleaved Caspase-3; Data are presented as mean ± SD (n = 3). Statistical analysis was performed using one-way ANOVA followed by Tukey’s *post hoc* test. **P < 0.01, compared with the control group. ^##^P < 0.01, compared with the model group.

## 4 Discussion

Ginsenosides are the principal bioactive constituents in *P. ginseng* and responsible for the *P. ginseng*’s wide range of pharmacological effects (Hwang et al., 2025). Ginsenosides have been demonstrated to modulate several putative biochemical markers shown to be important to the initiation and progression of PD. Evidence indicates that the key pharmacological actions of ginsenosides are associated with the antioxidant effects (Sng et al., 2022), improvement of mitochondrial dysfunction (Zhao et al., 2024), blockade of toxin uptake (Jiang et al., 2025), anti-inflammatory actions (Yang X. et al., 2025), and regulation of expression of neurotrophic factors (Zhang et al., 2023). In this study, Rg3-Lips were prepared and evaluated in both *in vitro* and *in vivo* PD models. Rg3-Lips exhibited neuroprotective effects, enhancing cell viability, ameliorating dopaminergic neuronal damage, and improving motor function. Rg3-Lips activated the Nrf2/GCLM signaling pathway to exert antioxidant effects, resulting in increased GSH levels and reduced ROS accumulation. Rg3-Lips also inhibited apoptosis and stabilized mitochondrial function by modulating the Bcl-2/Bax/Caspase-3 pathway, conferring the protective benefits to dopaminergic neurons. Furthermore, Rg3-Lips also showed enhanced pharmacological effects over the active pharmaceutical ingredient.

Novel drug delivery systems, including liposomes, nanoparticles, micelles, implants, controlled-release osmotic pumps, targeted drug conjugates, open the door to develop herbal novel drug delivery systems. Novel drug delivery technologies have gained the importance to achieve modified delivery of herbal drugs by increasing bioavailability, minimizing drug degradation and loss, and preventing side effects (Aggarwal et al., 2025). Using film dispersion method, this study prepared and identified Rg3-Lips to investigate the protective effects of Rg3-Lips on PD models induced by MPP^+^ and MPTP *in vitro* and *in vivo*. The cell death induced by MPP^+^ is a classic method for studying dopaminergic degenerative PD models *in vitro*, and SH-SY5Y human neuroblastoma cells, which have many characteristics of human neurons, have become the recognized PD model (Lal et al., 2024). LDH release was selected as a marker of cellular injury because increased LDH release reflects disruption of cell membrane integrity and therefore provides an indicator of MPP^+^-induced cytotoxicity. In addition, MPTP is a toxin with specific toxic effects on dopaminergic neurons in the substantia nigra and striatum. The prepared mouse model of MPTP has become an important tool for studying PD, capable of simulating the clinical and pathological features of PD (Wang et al., 2023). The open field, pole, and rotarod tests were selected to evaluate motor and behavioral impairments, while TH immunostaining was used to assess the integrity of the nigrostriatal dopaminergic system. This study used SH-SY5Y cells injury induced by MPP^+^ and PD mice induced by MPTP as models to prove that Rg3-Lips can exert antioxidant stress and inhibit cell apoptosis by regulating the Nrf2/GCLM and Bcl-2/Bax/Caspase-3 signaling pathways, and has the property of reducing dopaminergic nerve damage.

Oxidative stress damage is one of the main causes of PD. It is caused by the imbalance of cellular redox activity, which leads to excessive production of ROS. These ROS can oxidize cell membrane lipids, modify proteins, attack cytoplasmic RNA, damage mitochondrial DNA, and ultimately lead to the imbalance of cellular homeostasis (Jia et al., 2025). ROS, SOD, and GSH were selected to assess oxidative stress and antioxidant capacity, while Nrf2 and its downstream protein GCLM were examined to evaluate the involvement of the Nrf2/GCLM antioxidant pathway. The results of the *in vitro* experiments in this study indicate that MPP^+^ exposure significantly increased intracellular ROS levels, decreased the contents of SOD and GSH, and weakened their antioxidant capacity. Moreover, the Western blot experimental results show that MPP^+^ exposure also reduced the levels of Nrf2 and its downstream antioxidant protein GCLM. After Rg3-Lips intervention treatment, it could significantly improve the weakened antioxidant capacity induced by MPP^+^. The results of the *in vivo* experiments indicate that after MPTP exposure, the motor coordination and balance ability of mice decreased, causing damage to the dopaminergic neurons in the substantia nigra and the dopaminergic nerve fibers in the striatum. In addition, the GSH contents in the substantia nigra and striatum of mice decreased. In line with the findings of *in vitro*, the expression levels of Nrf2 and its downstream antioxidant protein GCLM in the substantia nigra of mice also decreased. And Rg3-Lips treatment could alleviate the reduction of levels of Nrf2 and GCLM. Therefore, both *in vitro* and *in vivo* experiments have demonstrated that Rg3-Lips exerts its antioxidant effect through the Nrf2/GCLM signaling pathway.

Studies have shown that ROS plays a significant role in the apoptotic mechanism of neurotoxicity mediated by MPP^+^. The oxidative damage caused by MPP^+^ may partially lead to the opening of mitochondrial transport pores and the disruption of mitochondrial membrane potential (Prasertsuksri et al., 2023). The *in vitro* experimental results of this study indicate that MPP^+^ exposure leads to a decrease in the mitochondrial membrane potential level of cells, and the TUNEL staining also show that the apoptosis rate of the model group cells increases, the expression of anti-apoptotic protein Bcl-2 located on the mitochondrial membrane is downregulated, while the expression of pro-apoptotic protein Bax is upregulated, and ultimately, Cleaved Caspase-3 is activated, inducing cell apoptosis. Rg3-Lips intervention can significantly inhibit the apoptosis rate and effectively regulate the expression levels of these apoptosis-related proteins. The *in vivo* experiment shows that after MPTP exposure, the number of apoptotic cells in the substantia nigra of mice significantly increases, the levels of pro-apoptotic proteins Bax and Cleaved Caspase-3 are upregulated, and the expression of anti-apoptotic protein Bcl-2 is downregulated. After Rg3-Lips treatment, these abnormal changes in proteins can be inhibited. Both *in vitro* and *in vivo* experiments have shown that Rg3-Lips exerts its anti-apoptotic effect through the Bcl-2/Bax/Caspase-3 signaling pathway. However, the present findings appear to differ from previous studies reporting neuroprotective effects of free Gs-Rg3 in rotenone-induced PD models. This discrepancy may be partly attributed to differences in Rg3 formulation and dosage. In the present study, Rg3 was formulated as liposomes, which may improve its solubility, stability, and delivery. In addition, a relatively lower dose of Gs-Rg3 (2.5 mg/kg) was used in the present study compared with the doses used in previous studies (5–20 mg/kg). The lower dose was selected to evaluate whether the liposomal formulation could exert neuroprotective effects at a relatively low dose, thereby highlighting the potential advantage of Rg3-Lips in improving the efficacy of Gs-Rg3.

Although there are important discoveries revealed by the present studies, there are also some limitations. First, pharmacokinetics of Rg3-Lips was not evaluated. Therefore, the pharmacokinetics of Rg3-Lips is inferred from previous work. If comparative pharmacokinetic parameters after administration of the Rg3-Lips and active pharmaceutical ingredient were investigated and clarified that Rg3-Lips increased the C_max_, AUC, half-life, it will undoubtedly strengthen the conclusion of this work. Second, the brain distribution and blood-brain barrier penetration of Rg3-Lips or released Gs-Rg3 were not evaluated in the present study. Therefore, although Rg3-Lips exhibited neuroprotective effects, we cannot clearly make a conclusion whether Rg3-Lips or released Gs-Rg3 reaches target location of brain. Further studies are needed to clarify the brain distribution and blood-brain barrier penetration of Rg3-Lips and their relationship with the observed neuroprotective effects. Thirdly, this study investigated the neuroprotective mechanism of Rg3-Lips against PD, focusing on its antioxidant and anti-apoptotic effects. However, the research did not establish a link integrating these two aspects. To precisely define the protective mechanism of Rg3-Lips, future work should elucidate the causal relationship connecting oxidative stress and apoptosis in dopaminergic neurons.

In summary, Rg3-Lips can improve the cell damage induced by MPP^+^ and the motor dysfunction in PD mice induced by MPTP through its antioxidant and anti-apoptotic effects. The neuroprotective mechanism of Rg3-Lips is to activate the Nrf2/GCLM signaling pathway, enhance the antioxidant capacity, reduce the accumulation of ROS and oxidative damage, thereby protecting dopaminergic neurons from oxidative stress damage; at the same time, by regulating the Bcl-2/Bax/Caspase-3 signaling pathway, stabilizing the mitochondrial membrane potential, and inhibiting the apoptosis of dopaminergic neurons. These results demonstrate that Rg3-Lips exerts neuroprotective effects in experimental PD models, suggesting its potential neuroprotective and therapeutic effects in experimental PD models. Furthermore, Rg3-Lips led to improved pharmacological effects over the conventional active pharmaceutical ingredient.

## Data Availability

The raw data supporting the conclusions of this article will be made available by the authors, without undue reservation.
